# Deriving movement properties and the effect of the environment from the Brownian bridge movement model in monkeys and birds

**DOI:** 10.1186/s40462-015-0043-8

**Published:** 2015-06-15

**Authors:** Kevin Buchin, Stef Sijben, E Emiel van Loon, Nir Sapir, Stéphanie Mercier, T Jean Marie Arseneau, Erik P Willems

**Affiliations:** Department of Mathematics and Computer Science, Technical University Eindhoven, Eindhoven, The Netherlands; Faculty of Mathematics, Ruhr-Universität Bochum, Bochum, Germany; Computational Geo-Ecology, Institute for Biodiversity and Ecosystem Dynamics, University of Amsterdam, Amsterdam, The Netherlands; Department of Evolutionary and Environmental Biology, The University of Haifa, Haifa, Israel; Institut de Biologie, Université de Neuchâtel, Neuchâtel, Switzerland; Anthropological Institute & Museum, University of Zurich, Zurich, Switzerland

**Keywords:** Brownian bridge movement model, Movement speed, Spatial distribution, Home range utilization, Migratory flight behaviour

## Abstract

**Background:**

The Brownian bridge movement model (BBMM) provides a biologically sound approximation of the movement path of an animal based on discrete location data, and is a powerful method to quantify utilization distributions. Computing the utilization distribution based on the BBMM while calculating movement parameters directly from the location data, may result in inconsistent and misleading results. We show how the BBMM can be extended to also calculate derived movement parameters. Furthermore we demonstrate how to integrate environmental context into a BBMM-based analysis.

**Results:**

We develop a computational framework to analyze animal movement based on the BBMM. In particular, we demonstrate how a derived movement parameter (relative speed) and its spatial distribution can be calculated in the BBMM. We show how to integrate our framework with the conceptual framework of the movement ecology paradigm in two related but acutely different ways, focusing on the influence that the environment has on animal movement. First, we demonstrate an *a posteriori* approach, in which the spatial distribution of average relative movement speed as obtained from a “contextually naïve” model is related to the local vegetation structure within the monthly ranging area of a group of wild vervet monkeys. Without a model like the BBMM it would not be possible to estimate such a spatial distribution of a parameter in a sound way. Second, we introduce an *a priori* approach in which atmospheric information is used to calculate a crucial parameter of the BBMM to investigate flight properties of migrating bee-eaters. This analysis shows significant differences in the characteristics of flight modes, which would have not been detected without using the BBMM.

**Conclusions:**

Our algorithm is the first of its kind to allow BBMM-based computation of movement parameters beyond the utilization distribution, and we present two case studies that demonstrate two fundamentally different ways in which our algorithm can be applied to estimate the spatial distribution of average relative movement speed, while interpreting it in a biologically meaningful manner, across a wide range of environmental scenarios and ecological contexts. Therefore movement parameters derived from the BBMM can provide a powerful method for movement ecology research.

**Electronic supplementary material:**

The online version of this article (doi:10.1186/s40462-015-0043-8) contains supplementary material, which is available to authorized users.

## Background

**Fig. 1:**
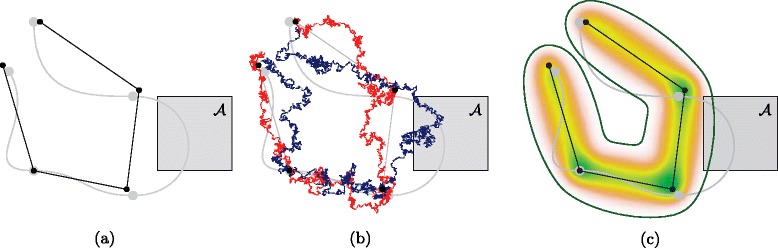
Linear interpolation compared to Brownian bridges. Linear interpolation compared to Brownian bridges. In this example the movement path is shown in gray and the location data as black dots connected by straight line segments. **a** Linear interpolation would incorrectly report that the movement path does not traverse the area $\mathcal {A}$. **b** Two realizations in the BBMM, one of which traverses $\mathcal {A}$. **c** Utilization distribution (density indicated by shading) and 99 % volume isopleth, which intersects $\mathcal {A}$

Modelling movement as a stochastic process provides means to estimate paths or location distributions when observations were not recorded continuously. This perspective is, however, often overlooked when analyzing movement based on discrete observations. For instance kernel-density estimation, which is frequently applied to movement data, does not take temporal autocorrelation into account. It is used for home-range estimation [1, 2] when the sampling rate is sufficiently low so that independence between observations can reasonably be assumed. Similarly, home range estimation based on minimum convex polygons [3] also ignores the actual movement between different locations. In other uses of movement data, locations are interpolated under the assumption of a linear movement path between observations [4]. This assumption is unrealistic except for densely sampled data, and can lead to wrong conclusions on sparser data as illustrated in Fig. 1(a).

Stochastic models like state-space models [5–7] and the *Brownian bridge movement model (BBMM)* [8–12] have been successfully applied for estimating the movement path and intensity of space use based on discrete location data. In this paper we explicitly focus on the BBMM (but see online Additional file 1 for a more elaborate discussion of the similarities and differences between the BBMM and state-space models). The BBMM takes the movement of animals into account to calculate space use patterns. It does so making relatively few assumptions, yet still making biological sense in that its parameters reflect real properties of the relocation data: measurement accuracy and –in a way– speed and directionality of movement. The assumption underlying the BBMM is that the entity exhibits purely random (i.e., Brownian)motion. In a typical scenario in which the BBMM is applied, we have multiple location measurements and are interested to infer the location at times in the interval between two consecutive measurements. Therefore, we condition Brownian motion on the measured locations at the observation times. Such a conditioned Brownian motion is called a Brownian bridge, which is illustrated in Fig. 1(b–c). The BBMM has the desirable property of being able to take measurement uncertainty into account, usually by assuming that this uncertainty follows aGaussian distribution around a given relocation point (which is an appropriate assumption for e.g. relocations obtained from GPS-telemetry [13]). In contrast to pure Brownian motion, however, additional Gaussian noise results in a process that is not Markov [14].

The use of the BBMM in the context of movement ecology was proposed by Bullard [8] and Horne et al. [9] and is defined by the measurement error and the *diffusion coefficient*, which relates to an organism’s mobility. Horne et al. propose to compute the diffusion coefficient using maximum likelihood estimation, thereby explicitly assuming homogeneous movement throughout an entire trajectory. However, as movement parameters change over time, it is biologically more realistic to allow the diffusion coefficient to vary. Kranstauber et al. [10] use the Bayesian information criterion to detect changes in the movement state of an organism, and use this to vary the diffusion coefficient over time. Bivariate Gaussian bridges factorise diffusion into a parallel and an orthogonal component [11]. A related algorithm is the *Biased random walk* proposed by Benhamou [15]. In his study the sampling density is increased using linear interpolation and then kernel density estimation is used at the resulting set of locations. Overall, these methods provide a more advanced estimate for the location distribution in relation to using a fixed diffusion coefficient, because they are more dynamic or segment-specific.

The BBMM has so far been exclusively used to compute utilization distributions. The analysis of movement, however, often does not ask for location as such, but rather focuses on derived movement parameters like relative speed, or more complex analysis tasks like similarity estimation between trajectories. In recent work Buchin et al. [16] show how to derive such parameters and how to perform fundamental analysis tasks under the assumption of a BBMM. Since their paper focused on the technical side of mathematically deriving the corresponding parameters, they assumed that movement takes place in a featureless space, not taking into account the external and internal factors that govern organismal movement.

Clearly though, these factors are essential for a proper biological understanding of animal movement. Nathan et al. [17] proposed a paradigm, which incorporates four basic components that affect a movement path: external factors, the internal state of the moving organism, its navigational capacity and its motion capacity. Getz and Saltz [18] present a framework for generating and analyzing movement paths using this paradigm, which can be used to generate movement paths by simulation and to segment movement paths by state-space methods. It does not, however, deal with the interpolation of location observations.

In this article we present a computational framework for movement analysis using the BBMM in the context of the movement ecology paradigm. Unique to our framework is the application of the BBMM beyond the estimation of utilization distributions to also calculate derived movement parameters and their spatial distribution. The derived movement parameter we focus on in this paper is relative speed and its spatial distribution. It is important to note that in the BBMM speed estimations necessarily need to be relative to a time scale, since Brownian motion is nowhere differentiable. Therefore, speed calculated in our framework is always *relative speed*^1^ and not an absolute measure. Further, we note that in our framework calculations are performed per bridge, and for any given bridge only the two adjacent observations are used. While this is in line with the work of Horne *et al* [9], this does not account for sequence of observations being not Markov [14] in the presence of measurement errors.

In the Results section we first discuss how various factors influencing a movement path can be incorporated in such an analysis. We differentiate between two related but acutely different approaches to do so. The first approach takes factors into account *a posteriori*, that is, they do not influence the movement model but are used to biologically interpret its outcome. The second takes factors into account *a priori*, that is, factors influence a key model parameter (the diffusion coefficient), and thereby the estimation of the movement path and derived properties.

We demonstrate our framework on data of two species with distinctly different movement.

We apply the *a posteriori* approach in a case study on how the movement speed of vervet monkeys (*Chlorocebus pygerythrus*) within a monthly ranging area is related to local vegetation density, whereas for the *a priori* approach we look at the flight mode of European bee-eaters (*Merops apiaster*) during migration.

## Results and discussion

### Computational aspects of the movement ecology framework

Organismal movement can be perceived as the outcome of the interaction between four key biological components: factors external to the organism, the organism’s internal state, its navigational capacity, and its motion capacity [17]. In this paper we focus on external factors and consider two ways in which their relation to the movement can be investigated. First we consider the case in which the components do not affect the computation of the BBMM, but instead are used *a posteriori* to biologically interpret its outcome. Second, we use the components *a priori* to dynamically modify a key parameter of the BBMM, the diffusion coefficient. This approach is in general more difficult to handle computationally. The aspect which dictates this difficulty is the degree of spatial dependence of the components. If they are independent of space, possibly conditional on time or some measurement (e.g. behaviour, which may be identified in the basis of a short acceleration signal) [19]), it can be handled in an analytical movement model. In contrast, if a factor is especially spatially dependent (e.g. a highly heterogeneous habitat), an explicit simulation of the spatial trajectory is required. This would effectively imply a multitude of simulations because we are interested in conditional distributions. If a factor is only varying relatively little over the length of a trajectory segment (e.g. atmospheric variables like wind or thermal convection), it is possible to make a quasi-steady state assumption and consider it as constant within a local spatial domain. This makes it much easier to handle spatial dependency in a BBMM.

In the following, we elaborate on the various settings at the hand of two case studies. In the first study the external factor (vegetation density) is given as raster data and has a strong spatial dependency. In this setting the *a posteriori* approach is applicable. The challenge here is to compute a spatial distribution of average speed. In the second study the external factor (atmospheric conditions) is given along the movement path and therefore the *a priori* approach is applicable. Since in this case study the movement behaviour depends crucially on the atmospheric conditions, the *a posteriori* approach would likely not provide added value.

### Movement speed of vervet monkeys – the *a posteriori* approach

**Fig. 2:**
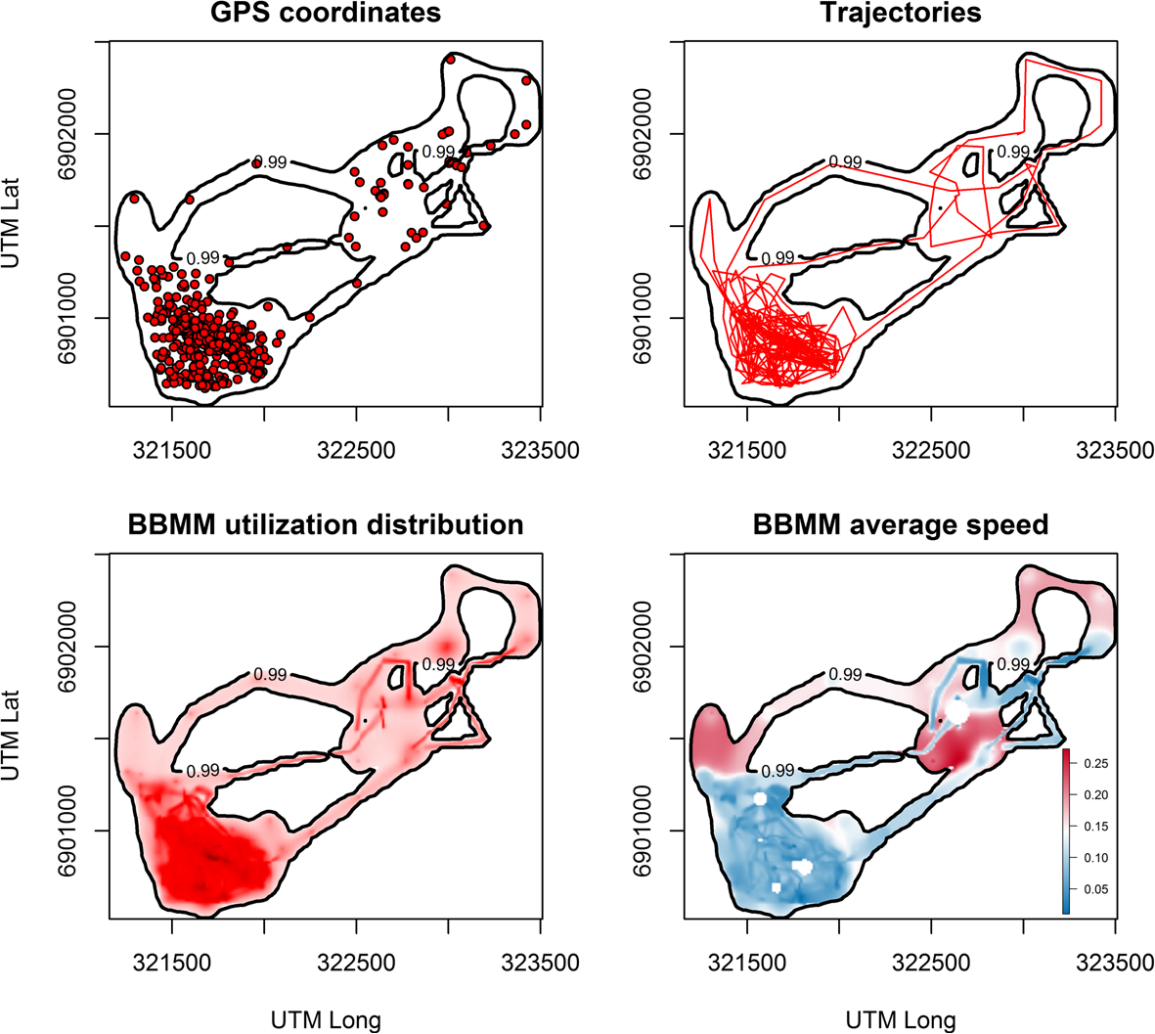
Spatial distribution of vervet monkey movement data. The Brownian bridge movement model takes the GPS fixes along the trajectories as input and is used to calculate a probability density distribution function of location (i.e. the utilisation distribution), but also a spatial distribution of a movement property like speed (red equals low, violet high speed). The black outline demarcates the 99 % volume isopleth

In the first case study, we apply our framework to investigate local differences in the movement speed of a wild group of free-ranging vervet monkeys within their ranging area over a 1 month period. Movement data were obtained from a GPS logger, deployed on a single adult female within the group and programmed to collect coordinates at hourly intervals during the animals’ daily activity period. In total, 465 relocations were collected this way (Fig. 2a), representing 31 daily trajectories (Fig. 2b). The GPS data is provided as Additional file 2.

We first employ our implementation of the dynamic BBMM to calculate the monthly utilization distribution of the monkeys and delineate their ranging area by a 99 % volume isopleth (Fig. 2c). This revealed the monkeys used an area of 1.3 km ^2^ over the observation period. Then we investigate how speed estimates from this dynamic BBMM relate to the external environment in which the animals are moving. We hypothesize that the monkeys travel faster in the more open, less densely vegetated areas of their range (due to greater exposure to predators and lower food availability), and slower in those areas in which the vegetation is more lush (more safety and food). We investigate this hypothesis by relating our average speed estimate (calculated over 5 minute time intervals; Fig. 2d) to local vegetation density, proxied by a high resolution (0.50 × 0.50 m ^2^) Normalized Difference Vegetation Index (NDVI) image (see Methods section). High NDVI values correspond to high vegetation density, whereas low values reflect sparse vegetation. We thus predict a negative association between the average movement speed of the monkeys and local NDVI values.

To statistically test this prediction, we generated 1000 random sample locations throughout the monthly range of the animals and extracted both local NDVI and speed estimate values. Since data exhibited significant levels of spatial autocorrelation (as indicated by inspections of Moran’s *I* values and correlograms), statistical significance of the association between local vegetationdensity and speed of movement was assessed using geographically effective degrees of freedom [20]. This analysis revealed a significant, negative correlation between local NDVI-values and BBMM-estimated average relative speed (*r*_*Pearson*_=−0.213,*F*_(1, 975.68)_=46.15,*p*<0.0001), in line with our biological expectations. We also performed the same analysis using only one diffusion coefficient (i.e., non-dynamic BBMM), which also showed a significant, negative correlation (*r*_*Pearson*_=−0.175,*F*_(1, 150.27)_=4.78,*p*=0.03).

### Migration of European bee-eaters – the *a priori* approach

The European bee-eater is a species that uses both flapping and soaring-gliding flight during its migratory movement. In this case study we use the relationship between atmospheric conditions and flight mode in this species [21*,*^22^] to construct a biologically informed BBMM that generates estimates of flight speed and trajectory uncertainty over different segments of the movement path, depending on likely flight-mode. Even though the influence of atmospheric conditions on the movement path (mediated by flight mode) has previously been investigated [22*–*25], this information has not yet been integrated into a movement model for the European bee-eater.

We hypothesize that soaring-gliding flight is characterized by an overall less straight, more tortuos path because in this flight mode birds may rely on the spatial variability of convective thermal intensity. Since soaring-gliding birds may actively select to circle in strong thermals that are not necessarily found in the exact direction of their flight destination, their path may be less direct or straight. Additionally, since migration speed scales differently with bird size for flapping and soaring-gliding flight modes, for relatively small birds like the European Bee-eater (mean body mass of 56 g [22]), it has been suggested that soaring-gliding will be slower than flapping flight [26]. To investigate these hypotheses we calculate and compare the diffusion coefficients and average flight speeds for the two flight modes using the BBMM.

**Fig. 3:**
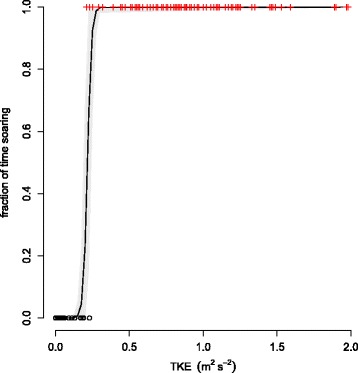
Logistic function. The logistic function describing the fraction of time the birds flew using soaring-gliding as a function of turbulence kinetic energy (TKE). The grey-shaded range is a 0.95 confidence interval

The data set consisted of 91, 141 and 94 segments characterized by flapping, mixed and soaring-gliding flight modes respectively (see [22] for additional details). The data was collected by radio telemetry, resulting in an irregular measurement frequency of approximately 6 minutes (343 seconds with standard deviation of 547 seconds). The data set is provided as Additional file 3. We use a model to predict the fraction of time spent on soaring-gliding flight as a function of atmospheric conditions (most notably, the magnitude of the Turbulence Kinetic Energy, or TKE). After calibration, our model classified the animals’ flight mode with an overall error rate of 1.1 %. This model has the following form:

$$\frac{e^{a\cdot \textrm{TKE}-b}}{1+e^{a \cdot \textrm{TKE} - b}}, $$

where the value (with 95 % confidence bounds) for parameter *a* is 74 (25 - 227), and for parameter *b* is 16 (5 - 50). Figure 3 shows the shape of this model as well as its predictive uncertainty.

**Fig. 4:**
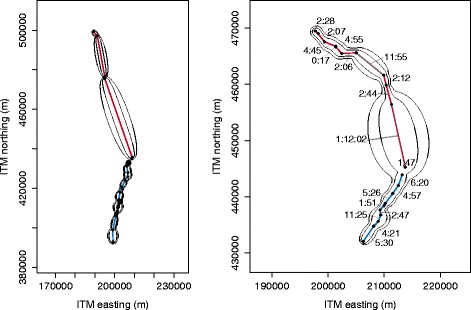
Changing diffusion coefficients. Two examples of the effect of a changing diffusion coefficient on the predicted trajectory. The coloured line is interpolated linearly between measured locations, where blue means a low diffusion coefficient mainly flapping flight), and red means a high diffusion coefficient (mainly soaring/gliding flight). The contours indicate the 90 % and 99 % volume isopleths based on the trajectory. In the example to the right the time passed between two measurements is indicated. A larger diffusion coefficient results in a wider contour. For instance, of two bridges of similar duration (4:55 and 4:57 minutes and length the red bridge has a wider contour than the blue

We selected the movement segments with the pure flapping and soaring-gliding flight modes and applied the maximum likelihood estimation by Horne et al. [9] separately to these. This resulted in estimated diffusion coefficients for flapping as 2965 m ^2^/s and 4505 m ^2^/s for soaring-gliding. This confirms our hypothesis that soaring-gliding is associated with a more tortuous flight path. The fact that this hypothesis could be investigated empirically on the basis of such sparse and irregularly sampled data is a distinct advantage of our approach over previous BBMM-based methods that, moreover are restricted to calculations of space use only. The difference in diffusion coefficients between the two flight modes is illustrated in Fig. 4. In this figure, the spatial distributions of two individuals are shown along with their flight mode. The movement path is clearly wider for segments with soaring-gliding flight than for those with flapping flight, and, to our knowledge, this aspect of flight mode on the migratory track has not yet been described elsewhere.

We calculated the movement speeds using our BBMM over 5 minute instances. Reasons for this resolution were the resolution of the original observations (approximately 6 minutes on average) and the fact that autocorrelation is very limited at this 5 minute resolution. At this resolution we found that the average relative cross-country speed for flapping flight was 9.7 m/s, while in soaring-gliding flight it was 8.5 m/s, a significant difference of 1.2 m/s (Welch two-sample T-test; 95 % confidence-interval: 0.91 - 1.56). The variance of relative cross-country speed for flapping flight was 16.2 m ^2^/s ^2^ and 7.1m ^2^/s ^2^ for soaring-gliding flight, a ratio of 2.30 (significant according to a 2-sided F-test; 95 % confidence-interval: 2.05 - 2.60).

**Fig. 5:**
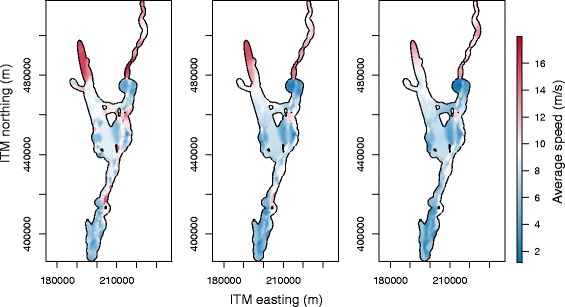
Spatial distribution of speed. Spatial distribution of speed of bee-eaters at different time scales, clipped to the 99 % volume isopleth using Israeli Transverse Mercator as coordinate grid. From left to right: 5 minutes, 15 minutes, and 30 minutes

With respect to the speed difference we note, though, that wind conditions were somewhat different between segments flown using different flight modes. For example, Sapir et al. [27] have recently found that bee-eaters undertaking flapping flight experienced higher headwinds, while during soaring-gliding wind was overall less intense and this may have influenced our calculations that dealt only with the cross-country flight speed. Figure 5 shows the spatial distribution of average cross-country speed relative to three different time scales. We further note, that the speed variability within the soaring-gliding flight mode could be resolved if fine-resolution observations (e.g. <30 seconds) would be available. In that scenario, the variability in speed differences which is now implicit in the higher diffusion coefficient for that mode would become explicit through a higher variance in speed (at fine resolutions) for the soaring-gliding flight mode. We further note that the calculated speeds depend on the diffusion coefficient, the displacement between two observations and the chosen time scale; therefore –as is the case here– a higher diffusion coefficient does not necessarily imply higher speed.

## Conclusions

We demonstrated how the Brownian bridge movement model can be extended to compute the spatial distribution of derived movement parameters, such as relative speed, and used two case studies to illustrate different ways (the *a posteriori* and *a priori* approach) in which our computational framework can integrate environmental factors with the BBMM. In both case studies our framework provided meaningful biological insights that could not have been obtained previously from the BBMM.

In the first case study, we used our framework to first calculate the utilization distribution and monthly ranging area of a group of vervet monkeys. Subsequently, we could analytically confirm the hypothesized relationship between the local average speed with which the animals traverse their ranging area to local vegetation density. Correlating local average speed to vegetation density required BBMM-based calculations novel to our paper, specifically an estimation of the spatial distribution of speeds. It would be interesting to see how a correlating variable could be used to estimate diffusion coefficients of a BBMM directly, which however seems like a computationally challenging task; this could mean that an *a posteriori* approach would be used as inspiration to apply an *a priori* approach.

In the second case study, we used existing knowledge about the relationship between atmospheric conditions and flight mode of migrating European bee-eaters, to evaluate whether different flight modes result in different average cross-country flight speed and tortuosity of the movement path. This was not possible in previous studies [21,22] due to varying sampling intervals. Here, however, we first fit a biologically informed BBMM, which then enabled us to demonstrate that soaring-gliding flight involves higher variability in route straightness and lower flight speeds than flapping flight. Our work therefore adds a novel perspective to bee-eater biology, and the novel findings –not discovered by the traditional approaches– demonstrate the usefulness of the new approach.

Both case studies heavily rely on the ability to not only estimate the spatial distribution of an animal but to also estimate derived movement parameters and their spatial distribution based on the BBMM – an application of the BBMM unique to our work. We note that many of the conceptual questions we address for the BBMM –like the use of spatial distributions of movement parameters to integrate environmental factors into the analysis– are also relevant to other movement models.

In general, our framework may apply to settings where environmental factors are expected to influence velocity. For terrestrial, aquatic and airborne organisms that could respectively be terrain ruggedness, currents and wind. However, also an organism’s internal state or interaction with other organisms may (when observations on these variables are available) be incorporated in the analysis. Even though our case studies do not represent all these possibilities, they do demonstrate that the derivation of movement parameters and their spatial distribution via BBMM is a powerful method for movement research.

## Methods

### Methods for computing movement parameters in the Brownian bridge movement model

We first discuss how various movement parameters are calculated in the BBMM and similar models. We then provide details on the specific methods used in the two case studies. The BBMM assumes that an entity exhibits Brownian motion between measured locations. A Brownianbridge is the distribution of this process conditioned on the locations of both endpoints. To model uncertainty in the measured locations and to avoid a degenerate probability distribution at the time of a measurement, the locations are often assumed to be normally distributed around the measured location. All of the following calculations are performed for individual Brownian bridges and only use the directly adjacent measurements. Note that in the presence of measurement errors the sequence of observations does not satisfy the Markov property [14], and any Brownian bridge actually depends on more than just the adjacent measurements. Thus, we need to assume that the measurement error is small relative to the diffusion coefficient.

If we assume that we have two locations ***x***_*i*_, ***x***_*i*+1_ measured at times *t*_*i*_, *t*_*i*+1_ with variances ${\delta _{i}^{2}}$ and $\delta _{i+1}^{2}$ respectively, the position ***X***_*t*_ at a time *t*∈[*t*_*i*_,*t*_*i*+1_] follows a circular bivariate normal distribution with parameters

$$\begin{array}{@{}rcl@{}} \boldsymbol{\mu}(t) &=& (1-\alpha) \boldsymbol{x}_{i} + \alpha \boldsymbol{x}_{i+1}, \\ \sigma^{2}(t) &=& (t_{i+1}-t_{i}) \alpha(1-\alpha) D + (1-\alpha)^{2} {\delta_{i}^{2}} + \alpha^{2} \delta_{i+1}^{2}. \end{array} $$

Here, $\alpha = \frac {t-t_{i}}{t_{i+1}-t_{i}}$ is a variable that linearly moves from 0 to 1 as *t* moves from *t*_*i*_ to *t*_*i*+1_ and *D* is the diffusion coefficient of the Brownian motion, which is often estimated by a maximum likelihood method [9]. When the trajectory contains different movement states over time, it may be appropriate to vary the diffusion over time rather than to keep it constant [10].

Given these probability distributions, derived parameters such as distance or speed (relative to a time scale) can be determined [16]. These parameters are important building blocks for the detection of many movement patterns. We summarize the results on the distributions of these parameters here, for full derivations we refer to [16] and online Additional file 4. Note that the derivation of velocity in [16] does not handle all possible dependencies and is superseded by the derivation in Appendix 1.

If the positions of two animals *A* and *B* at time *t* have independent circular normal distributions with means ***μ***_*A*_(*t*) and ***μ***_*B*_(*t*) and variances ${\sigma _{A}^{2}}(t)$ and ${\sigma _{B}^{2}}(t)$ respectively, the distance between *A* and *B* has a Rice distribution with parameters |***μ***_*A*_(*t*)−***μ***_*B*_(*t*)| and $\sqrt {{\sigma _{A}^{2}}(t) + {\sigma _{B}^{2}}(t)}$. The average velocity over a time interval [*t*_1_,*t*_2_] is given by the difference between two (generally not independent) circular normal distributions, for ***X***(*t*_2_) and ***X***(*t*_1_). The velocity has a circular normal distribution with mean $\frac {\boldsymbol {\mu }(t_{2})-\boldsymbol {\mu }(t_{1})}{t_{2}-t_{1}}$, while the expression for the variance depends on the number of location measurements that were obtained between *t*_1_ and *t*_2_.

Let *t*_*s*_, *t*_*i*_ and *t*_*f*_ be the time stamps of three consecutive observations with location variances ${\delta _{s}^{2}}$, ${\delta _{i}^{2}}$ and ${\delta _{f}^{2}}$ respectively, chosen such that *t*_*s*_≤*t*_1_<*t*_*i*_. The observation at *t*_*f*_ is only needed in the calculations if *t*_*i*_<*t*_2_≤*t*_*f*_. The variance of the velocity is:

$$ {\sigma_{V}^{2}} (t_{1}, t_{2}) =\left\{\!\! \begin{array}{ll} \frac{{\delta_{s}^{2}} + {\delta_{i}^{2}}}{(t_{i}-t_{s})^{2}} + \left(\frac{1}{t_{i}-t_{s}} + \frac{1}{t_{2}-t_{1}}\right) & \text{if}\; t_{1} < t_{2} \leq t_{i},\\ \vspace*{9pt} \frac{\sigma^{2}(t_{1}) + \sigma^{2}(t_{2}) - 2\left(\frac{t_{1}-t_{s}}{t_{i}-t_{s}}\right)\left(\frac{t_{f}-t_{2}}{t_{f}-t_{i}}\right){\delta_{i}^{2}}}{(t_{2}-t_{1})^{2}} & \text{if \(t_{i} < t_{2} \leq t_{f}\),}\\ \vspace*{6pt} \frac{\sigma^{2}(t_{1}) + \sigma^{2}(t_{2})}{(t_{2}-t_{1})^{2}} & \text{otherwise.} \end{array} \right. $$

Let ***μ***_*V*_ and ${\sigma _{V}^{2}}$ be the parameters of the velocity distribution over a time interval [*t*_1_,*t*_2_]. Speed (the absolute value of velocity) over this interval then has a Rice distribution with parameters |***μ***_*V*_| and *σ*_*V*_. The direction of this velocity has a distribution with density

$$\begin{aligned} f(\gamma) =&\, \frac{e^{-\frac{\nu^{2}}{2}}}{2\pi} + \frac{\nu\cos\eta}{2\sqrt{2\pi}} \exp\left(\frac{\nu^{2}\left(\cos^{2}\eta-1\right)}{2}\right)\\ &\times\left(1+\text{erf}\left(\frac{\nu\cos\eta}{\sqrt{2}}\right)\right), \end{aligned} $$

where $\nu = \frac {|\boldsymbol {\mu }_{V}|}{\sigma _{V}}$ is the noncentrality of the velocity distribution and *η*=atan2(***μ***_*V*_)−*γ* is the angle between the direction of the mean and the direction under consideration.

To obtain spatial distributions of speed, we consider the speed over a time interval [*t*+*Δt*_*s*_,*t*+*Δt*_*f*_], after fixing the position at one time *t* to a fixed location. If the time interval contains the time at which the position is fixed, i.e. *Δt*_*s*_≤0 and *Δt*_*f*_≥0, the position distributions at both endpoints of the interval are independent. The conditioned velocity and speed distributions are then determined from these two distributions. The spatial distribution of speed and the effect of the choice of the time scale (*Δt*_*f*_−*Δt*_*s*_) is illustrated in Fig. 5 by the example of the data used in the second case study.

We do not give the details about these position distributions here, but refer to Appendix 1. Let ***μ***_*s*_, ***μ***_*f*_, ${\sigma _{s}^{2}}$ and ${\sigma _{f}^{s}}$ represent the respective means and variances of the conditioned positions at both endpoints of the interval. Then by independence of the positions the velocity distribution conditioned on ***X***_*t*_=***x*** is given by

$${\fontsize{9.3}{6}\begin{aligned} \boldsymbol{V}_{\boldsymbol{x}; t} (t+\Delta t_{s}, t+\Delta t_{f}) &= \frac{\boldsymbol{X}_{t+\Delta t_{f}} - \boldsymbol{X}_{t+\Delta t_{s}}}{\Delta t_{f} - \Delta t_{s}}\\ &\sim \mathcal{N}\left(\frac{\boldsymbol{\mu}_{f} - \boldsymbol{\mu}_{s}}{\Delta t_{f} - \Delta t_{s}}\right)\left(\frac{{\sigma_{s}^{2}} + {\sigma_{f}^{2}}}{\left(\Delta t_{f} - \Delta t_{s}\right)^{2}}\right). \end{aligned}} $$

As discussed before, the speed has a Rice distribution. We determine the average speed at a particular location by computing a weighted average over time of the mean speed. The weight is given by the probability density of the animal’s position at the given time and location. That is,

(1) $$  S(\boldsymbol{x}) = \frac{1}{\int\! f_{\boldsymbol{X}{_{t}}}({\boldsymbol{x}})\,dt} \int f\boldsymbol{X}_{t}({\boldsymbol{x}}) \mathbb{E}\left[|\boldsymbol{V}_{\boldsymbol{x};t}(t+\Delta t_{s}, t+\Delta t_{f})|\right] dt.  $$

### Methods for the analysis of the movement speed of vervet monkeys in relation to their environment

Vervet monkeys are group-living primates that are abundant throughout most of sub-Saharan Africa [28]. They occur in stable, mixed-sex groups of typically 25-30 animals that consist of multiple adult males and females along with their offspring. Patterns of home range selection and general space use are strongly affected by external environmental factors such as primary productivity and vegetation structure [29] as well as the distribution of food, surface water and perceived predation risk [30].

In order to investigate whether the movement speed of animals is similarly affected by external variables, the data used in this case study were collected on a wild group of vervet monkey ranging freely in their natural habitat in Kwazulu-Natal, South Africa, during December2010. A digital telemetry collar (e-obs Type 1A, 69 gper unit, equivalent to just over 2 % of the tagged animal’s body weight; All work at the Inkawu Vervet project was approved by the relevant local authorities (the ethical boards of Ezemvelo KwaZulu-Natal Wildlife and the University of Cape Town, South Africa), and complies with EU-directive 2010/63/EU on the protection of animals used for scientific purposes) was deployed on a single adult female within the group and programmed to obtain GPS-fixes at hourly intervals throughout the daily activity phase of the animals (05:00 – 19:00). Given that vervet monkey groups typically move as coherent units through the landscape, GPS-coordinates obtained from the tagged female were taken to represent the movement of the entire group. Local vegetation density was estimated from a multi-spectral, high-resolution (0.50 × 0.50 m ^2^ pixel size) satellite image (WorldView II, DigitalGlobe Inc.) obtained over the study-period. From this image, we calculated the Normalized Difference Vegetation Index (NDVI) [31], a well-established spectral correlate of primary productivity and vegetation structure.

In our dynamic BBMM calculations, we did not consider bridges at the beginning of the day that stayed very close (≤50m) to the starting location, as this indicated the monkeys had not commenced moving yet, and similarly at the end of the day near the final location. On the remaining bridges the method by Kranstauber et al. [10] was used to estimate the diffusion coefficient (using a margin of 3 and a window size of 7). The average speed distribution presented in the Results section, was computed at a time scale *Δt* of 5 minutes. Mean speed was computed as defined in Equation 1, over two time intervals relative to the focal point: one directly preceding it (i.e. *Δt*_*s*_=−*Δt*, *Δt*_*f*_=0), and one directly following it (i.e. *Δt*_*s*_=0, *Δt*_*f*_=*Δt*). If we had used only one of these intervals, we would not have been able to compute a speed near the beginning or end of the daily activity period, which could have resulted in missing values in the distribution. For the analysis with only one diffusion coefficient we used the method by Horne et al. [9]. The R scripts that were used in this analysis are provided as Additional file 5.

### Methods for migration of European bee-eaters

This case study deals with the northward migration of the European bee-eater through the Arava Valley in southern Israel. The species is a very common passage migrant during both autumn and spring throughout the entire country [32]. In the 2005 and 2006 spring migration seasons, a total of 11 bee-eaters were trapped, marked and tagged with radio transmitters. Using portable systems, birds were followed over a total of 810 km during which their flight mode was established throug h both wing flap signals and the unique signature of circling flight in the recorded transmission (for details see [21,22]; Bee-eater trapping permits were obtained from the Israeli Nature and Parks Authority (permits 2005/22055, 2006/25555) and the experimental procedure was approved by the Animal Care and Use Committee of the Hebrew University of Jerusalem (permit NS-06-07-2)). Trajectories were annotated with simulated atmospheric conditions at appropriately short and small scales using the *Regional Atmospheric Modeling System* (RAMS; [33,34]). The relationship between bird flight mode (flapping, soaring-gliding and mixed flight) and atmospheric conditions are described in [22]. That study confirmed that turbulence kinetic energy (TKE, in m ^2^/s ^2^), as an indicator of convective updraught intensity in the atmosphere, facilitates soaring and gliding. In the current study, the relationship between bird flight mode and the movement path was estimated by calculating the effects of bird flight mode on the animal diffusion coefficient in the BBMM [16].

The relation between TKE and flight mode as well as between flight mode and the diffusion coefficient was determined by considering only movement stretches with flapping and pure soaring-gliding modes (hence omitting the mixed flight modes). The mixed flight mode is highly variable and biomechanically not as well defined as flapping or soaring-gliding flight.

A univariate logistic model was fitted to estimate the fraction of soaring flight (s) as a function of TKE. Model and parameter significance was tested for this model (using a 0.05 significance level), as well as the overall classification error. Subsequently, the BBMM was fitted to segments with flapping flight and soaring-gliding flight separately, resulting in estimates for the diffusion coefficients for each of these flight modes. Next, the diffusion coefficient for the mixed flight mode was estimated by weighting the two diffusion coefficients with the fraction of time spent in each flight mode:

$$D_{m} = (1-s) D_{f} + s\cdot D_{s}, $$

where *D*_*m*_, *D*_*f*_ and *D*_*s*_ refer to the diffusion coefficients of respectively mixed, flapping and soaring-gliding flight. The fraction *s* is obtained from the aforementioned logistic model. Using this parameterisation, the complete flight trajectories are estimated per bird by the BBMM.

In addition to the estimated model coefficients, the results of this analysis are presented in the form of probability maps of movement for selected individuals, showing not only the most likely movement path but also the uncertainty in this as a function of distance between observation points and flight mode (as illustrated in Fig. 4). The R scripts that were used in this analysis are provided as Additional file 6.

## Availability of supporting data

The vervet monkey GPS data set, the bee-eater data set, and the R scripts used in the analysis are included as additional files with the article.

## Endnote

^1^ For ease of readability we refer to relative speed simply as speed throughout the article.

## Additional files

Additional file 1
**The Brownian bridge movement model in relation to state-space models.** Document containing a discussion of the Brownian bridge movement model in relation to state-space models.

Additional file 2
**Vervet monkey data set.** GPS data set used in the first case study.

Additional file 3
**Bea-eater data set.** Data set used in the second case study.

Additional file 4
**Relative velocity in the Brownian bridge movement model.** Document containing the derivation of the distribution of relative velocity over time in the Brownian bridge movement model. From this we derive the distribution of speed and of direction and the spatial distribution of average speed.

Additional file 5
**R scripts (first study).** R scripts used in the first case study.

Additional file 6
**R scripts (second study).** R scripts used in the second case study.

## Abbreviations

BBMM: Brownian bridge movement model
GPS: Global Positioning System
RAMS: Regional Atmospheric Modeling System
TKE: Turbulence kinetic energy
