# Supplementary material for: Deriving movement properties and the effect of the environment from the Brownian bridge movement model in monkeys and birds
Source: Mov Ecol. 2015 Jun 15;3(1):18. doi: 10.1186/s40462-015-0043-8 (PMC4466871; doi:10.1186/s40462-015-0043-8)
Supplement: Additional file 1 — The Brownian bridge movement model in relation to state-space models. Document containing a discussion of the Brownian bridge movement model in relation to state-space models. [file 40462_2015_43_MOESM1_ESM.pdf]

ADDITIONAL FILE

The Brownian bridge movement model in relation to state-space models

Kevin Buchin[1*], Stef Sijben[2], E Emiel van Loon[3], Nir Sapir[4], Stéphanie Mercier[5], T Jean Marie Arseneau[6] and Erik P Willems[6]

*Correspondence: k.a.buchin@tue.nl
[1]Department of Mathematics and Computer Science, Technical University Eindhoven, Eindhoven, The Netherlands
Full list of author information is available at the end of the article

Content

We here discuss the Brownian bridge movement model in relation to state-space models. This is Additional file 1 of the paper *Deriving movement properties and the effect of the environment from the Brownian bridge movement model in monkeys and birds.*

Discussion

Similar to the Brownian bridge movement model (BBMM) discussed in the paper *Deriving movement properties and the effect of the environment from the Brownian bridge movement model in monkeys and birds*, it is possible to calculate the conditional uncertainty for location and velocity with state-space models (SSMs). SSMs form a very general class of stochastic differential equations that explicitly separate the process model from an observation model and can be used for prediction in an unconditional (estimating states in the absence of observations) as well as a conditional (estimation by taking observations into account) way (see [1] for a comprehensive overview and [2] for a concrete implementation).

In fact, SSMs can estimate more state variables than location and velocity (e.g. physiological and behavioural variables [2]) as long as process models are specified for these variables. When applying an SSM with conditional simulation there are essentially two modes: a) taking the observations up to a given point into account, and subsequently predicting ahead in time (filtering), and b) taking all observations in a relevant domain into account and reconstructing the most likely state (smoothing). Due to this generality, it is possible to specify a BBMM as a simple SSM. Hereby the process model would comprise random movement (i.e. constantly diffusive, with no bias towards a particular area), the observations would be without error, and the model would be applied as a smoother (because observations are present at both ends of an unknown trajectory). Sequential Monte Carlo methods [3] are used to apply filtering or smoothing to stochastic differential equations. These estimation techniques are however complex and computationally costly: when estimating basic Brownian motion with perfect observations, a BBMM formulation will be easier to implement than an equivalent state-space model.

When adding relations between environmental variables (or other relevant states) the differences between SSMs and BBMMs become less clear. In this setting the entire BBMM-analysis becomes more complex (but not the estimation of location and velocity through the BBMM per se). An advantage of SSMs over BBMMs is

that the errors and information associated with the additional states are propagated consistently throughout the model, provided that appropriate error models are defined. When applying BBMMs, the errors in the additional relations (both a priori or a posterior) are not automatically translated into the other states.

In Kranstauber et al. [4] as well as Byrne et al. [5] it is mentioned that SSMs may be able to describe movement with highly variable observation frequencies better than BBMMs. While we agree with this statement in general, we would like to add that in this case the SSM would not resemble the mechanism underlying BBMMs but specify mechanisms in addition to constrained Brownian motion.

Author details

[1]Department of Mathematics and Computer Science, Technical University Eindhoven, Eindhoven, The Netherlands. [2]Faculty of Mathematics, Ruhr-Universität Bochum, Bochum, Germany. [3]Computational Geo-Ecology, Institute for Biodiversity and Ecosystem Dynamics, University of Amsterdam, Amsterdam, The Netherlands. [4]Department of Evolutionary and Environmental Biology, The University of Haifa, Haifa, Israel. [5]Institut de Biologie, Université de Neuchâtel, Neuchâtel, Switzerland. [6]Anthropological Institute & Museum, University of Zurich, Zurich, Switzerland.

References

1. Patterson, T.A., Thomas, L., Wilcox, C., Ovaskainen, O., Matthiopoulos, J.: State-space models of individual animal movement. Trends Ecol. Evol. **23**, 87–94 (2008)
2. Dowd, M., Joy, R.: Estimating behavioral parameters in animal movement models using a state-augmented particle filter. Ecology **92**, 568–575 (2011). doi:10.1890/10-0611.1
3. Liu, J.S., Chen, R.: Sequential monte carlo methods for dynamic systems. Journal of the American Statistical Association **93**, 1032–1044 (1998)
4. Kranstauber, B., Kays, R., LaPoint, S.D., Wikelski, M., Safi, K.: A dynamic Brownian bridge movement model to estimate utilization distributions for heterogeneous animal movement. J. Anim. Ecol. **81**(4), 738–746 (2012). doi:10.1111/j.1365-2656.2012.01955.x
5. Byrne, M.E., J.C., M., Hinton, J.W., Chamberlain, M.J., B.A., C.: Using dynamic brownian bridge movement modelling to measure temporal patterns of habitat selection. Journal of Animal Ecology **13**, 1234–1243 (2014). doi:10.1111/1365-2656.12205
