# Supplementary material for: Deriving movement properties and the effect of the environment from the Brownian bridge movement model in monkeys and birds
Source: Mov Ecol. 2015 Jun 15;3(1):18. doi: 10.1186/s40462-015-0043-8 (PMC4466871; doi:10.1186/s40462-015-0043-8)
Supplement: Additional file 4 — Relative velocity in the Brownian bridge movement model. Document containing the derivation of the distribution of relative velocity over time in the Brownian bridge movement model. From this we derive the distribution of speed and of direction and the spatial distribution of average speed. [file 40462_2015_43_MOESM4_ESM.pdf]

ADDITIONAL FILE

Relative Velocity in the Brownian bridge movement model

Kevin Buchin[1*], Stef Sijben[2], E Emiel van Loon[3], Nir Sapir[4], Stéphanie Mercier[5], T Jean Marie Arseneau[6] and Erik P Willems[6]

*Correspondence: k.a.buchin@tue.nl
[1]Department of Mathematics and Computer Science, Technical University Eindhoven, Eindhoven, The Netherlands
Full list of author information is available at the end of the article

Content

We here give a derivation of the distribution of velocity in the Brownian bridge movement model relative to a given time scale. From this we further derive the distributions of speed and direction and of the spatial distribution of speed. This is Additional file 2 of the paper *Deriving movement properties and the effect of the environment from the Brownian bridge movement model in monkeys and birds.*

1 Brownian bridges

The Brownian bridge movement model (BBMM) assumes that an entity exhibits Brownian motion between measured locations. A Brownian motion can be characterized by a starting location $\boldsymbol{x}$ and a scale parameter σ_m^2, which is called the *diffusion coefficient*. If $\boldsymbol{X}_t$ is a Brownian motion with parameters $\boldsymbol{x}$ and σ_m^2, then its distribution is a (multivariate) normal distribution: $\boldsymbol{X}_t \sim \mathcal{N}\left(\boldsymbol{x}, t\sigma_m^2\right)$.

In the BBMM we have multiple location measurements and want to reason about the location at times between the times of these measurements. Therefore, we condition a Brownian motion on the location $\boldsymbol{X}_T$ at some time T, as well as the starting location $\boldsymbol{X}_0$. Such a conditioned Brownian motion is called a *Brownian bridge*. This results in the following distribution [1]:

$$\left(\boldsymbol{X}_t \middle| \boldsymbol{X}_0 = \boldsymbol{a} \wedge \boldsymbol{X}_T = \boldsymbol{b}\right) \sim \mathcal{N}\left((1-\alpha)\boldsymbol{a} + \alpha\boldsymbol{b}, T\alpha(1-\alpha)\sigma_m^2\right), \tag{1}$$

where $\alpha := \frac{t}{T}$.

To model uncertainty in the measured locations and to avoid degenerate probability distributions at the time of a measurement, the locations are often assumed to be independently normally distributed around the measured locations. If we assume that we have two locations $\boldsymbol{x}_{(i)}$, $\boldsymbol{x}_{(i+1)}$ measured at times t_i, t_{i+1} with variances δ_i^2 and δ_{i+1}^2 respectively, the position at a time $t \in [t_i, t_{i+1}]$ follows a multivariate normal distribution with parameters

$$\begin{aligned} \boldsymbol{\mu}_i(t) &= (1-\alpha)\boldsymbol{x}_i + \alpha\boldsymbol{x}_{i+1}, \\ \sigma_i^2(t) &= (1-\alpha)^2\delta_i^2 + \alpha^2\delta_{i+1}^2 + (t_{i+1} - t_i)\alpha(1-\alpha)\sigma_m^2, \end{aligned} \tag{2}$$

where $\alpha := \frac{t-t_i}{t_{i+1}-t_i}$. The following derivations are based on Equation 2. This equation uses only the measurements at the start and the end of the bridge. Note that in the presence of measurement errors also other measurements have a weak influence on the positions [2], which we do not include in the derivations.

2 Relative Velocity

Velocity is the rate of change of the location. Velocity is a vector quantity, while *speed* is the scalar absolute value of the velocity. Movement speed and direction together specify the velocity. Velocity (and therefore also speed and direction) are dependent on the temporal scale at which it is derived. We therefore derive a distribution for the average velocity relative to a given time interval. In an analysis the length of the time interval needs to be chosen according to the scale of the pattern considered and according to the goal of the analysis.

The distribution of the velocity depends on the number of location measurements in the interval of interest.[1]

2.1 No measurements in the interval.

Let $\boldsymbol{X}_s \sim \mathcal{N}\left(\boldsymbol{\mu}_s, \delta_s^2\right)$ and $\boldsymbol{X}_f \sim \mathcal{N}\left(\boldsymbol{\mu}_f, \delta_f^2\right)$ represent the positions at the endpoints of a Brownian bridge that were obtained at times t_s and t_f. If we want to determine the velocity distribution over a time interval $[t_1, t_2]$ such that $t_s \leq t_1 < t_2 \leq t_f$, the positions $\boldsymbol{X}_1, \boldsymbol{X}_2$ of an entity at these times cannot be regarded as independent. Instead, we fix $\boldsymbol{X}_1$ at a position $\boldsymbol{x}$ and then use the Markov property of Brownian motion to derive a distribution for $\boldsymbol{X}_2$ using Equation 1. We therefore obtain two Brownian bridges, one over $[t_s, t_f]$ parameterized by $\alpha = \frac{t_1 - t_s}{T}$ (where $T = t_f - t_s$), the other over $[t_1, t_f]$ parameterized by $\beta = \frac{t_2 - t_1}{t_f - t_1}$.

In the following, $\phi(\boldsymbol{x}; \boldsymbol{\mu}, \sigma^2)$ denotes the probability density function of a circular normal distribution with parameters $\boldsymbol{\mu}$ and σ^2, evaluated at $\boldsymbol{x}$, while $\phi(\boldsymbol{\mu}, \sigma^2)$ denotes this density function without evaluating it. $f_{\boldsymbol{X}}(\boldsymbol{x})$ denotes the probability density of a random variable $\boldsymbol{X}$, evaluated at $\boldsymbol{x}$. We integrate $\boldsymbol{X}_s$, $\boldsymbol{X}_1$ and $\boldsymbol{X}_f$ over all positions they can take (i.e. $\mathbb{R}^d$) to obtain the distribution of the displacement over this interval:

$$
\begin{aligned}
&f_{\boldsymbol{X}_2 - \boldsymbol{X}_1}(\boldsymbol{v}) \\
&= \int_{\mathbb{R}^d} \int_{\mathbb{R}^d} \int_{\mathbb{R}^d} f_{\boldsymbol{X}_s}(\boldsymbol{s})\, f_{\boldsymbol{X}_f}(\boldsymbol{f})\, f_{\boldsymbol{X}_1}\left(\boldsymbol{x} \middle| \boldsymbol{X}_s = \boldsymbol{s} \wedge \boldsymbol{X}_f = \boldsymbol{f}\right) \\
&\qquad\qquad f_{\boldsymbol{X}_2}\left(\boldsymbol{x} + \boldsymbol{v} \middle| \boldsymbol{X}_1 = \boldsymbol{x} \wedge \boldsymbol{X}_f = \boldsymbol{f}\right) d\boldsymbol{x}\, d\boldsymbol{f}\, d\boldsymbol{s} \\
&= \int_{\mathbb{R}^d} \int_{\mathbb{R}^d} \phi(\boldsymbol{s}; \boldsymbol{\mu}_s, \delta_s^2)\phi(\boldsymbol{f}; \boldsymbol{\mu}_f, \delta_f^2) \int_{\mathbb{R}^d} \phi(\boldsymbol{x}; (1-\alpha)\boldsymbol{s} + \alpha\boldsymbol{f}, T\alpha(1-\alpha)\sigma_m^2) \\
&\qquad\qquad \phi(\boldsymbol{x} + \boldsymbol{v}; (1-\beta)\boldsymbol{x} + \beta\boldsymbol{f}, (t_f - t_1)\beta(1-\beta)\sigma_m^2)\, d\boldsymbol{x}\, d\boldsymbol{f}\, d\boldsymbol{s}.
\end{aligned} \tag{3}
$$

We first evaluate the inner integral to obtain the distribution of $\boldsymbol{X}_2 - \boldsymbol{X}_1$ if $\boldsymbol{s}$ and $\boldsymbol{f}$ are fixed. We first rewrite the expression as a convolution and then use the property that the convolution of two normal densities is again the density of a normal distribution. Note that in this step we use $\frac{1-\beta}{\beta} = \frac{(t_f - t_1) - (t_2 - t_1)}{t_2 - t_1} = \frac{t_f - t_2}{t_2 - t_1}$.

[1]Note that the derivation of velocity given in [3] does not handle all cases and dependencies.

$$\begin{aligned}
&\int_{\mathbb{R}^d} \phi(\boldsymbol{x}; (1-\alpha)\boldsymbol{s} + \alpha\boldsymbol{f}, T\alpha(1-\alpha)\sigma_m^2) \\
&\qquad\qquad\qquad \phi(\boldsymbol{x}+\boldsymbol{v}; (1-\beta)\boldsymbol{x} + \beta\boldsymbol{f}, (t_f - t_1)\beta(1-\beta)\sigma_m^2) d\boldsymbol{x} \\
&= \int_{\mathbb{R}^d} \phi(\boldsymbol{x}; (1-\alpha)\boldsymbol{s} + \alpha\boldsymbol{f}, T\alpha(1-\alpha)\sigma_m^2)\phi(\beta\boldsymbol{x}; -\boldsymbol{v} + \beta\boldsymbol{f}, (t_f - t_1)\beta(1-\beta)\sigma_m^2) d\boldsymbol{x} \\
&= \int_{\mathbb{R}^d} \phi(\boldsymbol{x}; (1-\alpha)\boldsymbol{s} + \alpha\boldsymbol{f}, T\alpha(1-\alpha)\sigma_m^2)\phi\left(-\boldsymbol{x}; \frac{\boldsymbol{v}}{\beta} - \boldsymbol{f}, \frac{(t_f - t_1)(t_f - t_2)}{t_2 - t_1}\sigma_m^2\right) d\boldsymbol{x} \\
&= \left[\phi((1-\alpha)\boldsymbol{s} + \alpha\boldsymbol{f}, T\alpha(1-\alpha)\sigma_m^2) * \phi\left(\frac{\boldsymbol{v}}{\beta} - \boldsymbol{f}, \frac{(t_f - t_1)(t_f - t_2)}{t_2 - t_1}\sigma_m^2\right)\right](\boldsymbol{0}) \\
&= \phi\left(\boldsymbol{0}; (1-\alpha)(\boldsymbol{s} - \boldsymbol{f}) + \frac{\boldsymbol{v}}{\beta}, \left(T\alpha(1-\alpha) + \frac{(t_f - t_1)(t_f - t_2)}{t_2 - t_1}\right)\sigma_m^2\right).
\end{aligned}$$

Next we replace the inner integral of Equation 3 by this result and use the same technique to evaluate the middle integral:

$$\begin{aligned}
&\int_{\mathbb{R}^d} \phi(\boldsymbol{f}; \boldsymbol{\mu_f}, \delta_f^2) \\
&\qquad\qquad \phi\left(\boldsymbol{0}; (1-\alpha)(\boldsymbol{s} - \boldsymbol{f}) + \frac{\boldsymbol{v}}{\beta}, \left(T\alpha(1-\alpha) + \frac{(t_f - t_1)(t_f - t_2)}{t_2 - t_1}\right)\sigma_m^2\right) d\boldsymbol{f} \\
&= \int_{\mathbb{R}^d} \phi(\boldsymbol{f}; \boldsymbol{\mu}_f, \delta_f^2)\phi\left((\alpha - 1)(\boldsymbol{s} - \boldsymbol{f}); \frac{\boldsymbol{v}}{\beta}, \left(T\alpha(1-\alpha) + \frac{(t_f - t_1)(t_f - t_2)}{t_2 - t_1}\right)\sigma_m^2\right) d\boldsymbol{f} \\
&= \int_{\mathbb{R}^d} \phi(\boldsymbol{f}; \boldsymbol{\mu}_f, \delta_f^2)\phi\left(\boldsymbol{s} - \boldsymbol{f}; \frac{\boldsymbol{v}}{(\alpha-1)\beta}, \left(\frac{T\alpha}{1-\alpha} + \frac{(t_f - t_1)(t_f - t_2)}{(t_2 - t_1)(1-\alpha)^2}\right)\sigma_m^2\right) d\boldsymbol{f} \\
&= \int_{\mathbb{R}^d} \phi(\boldsymbol{f}; \boldsymbol{\mu}_f, \delta_f^2)\phi\left(\boldsymbol{s} - \boldsymbol{f}; -\frac{T}{t_2 - t_1}\boldsymbol{v}, \left(\frac{T\alpha}{1-\alpha} + \frac{(t_f - t_1)(t_f - t_2)}{(t_2 - t_1)(1-\alpha)^2}\right)\sigma_m^2\right) d\boldsymbol{f} \\
&= \left[\phi(\boldsymbol{\mu}_f, \delta_f^2) * \phi\left(-\frac{T}{t_2 - t_1}\boldsymbol{v}, \left(\frac{T\alpha}{1-\alpha} + \frac{(t_f - t_1)(t_f - t_2)}{(t_2 - t_1)(1-\alpha)^2}\right)\sigma_m^2\right)\right](\boldsymbol{s}) \\
&= \phi\left(\boldsymbol{s}; \boldsymbol{\mu}_f - \frac{T}{t_2 - t_1}\boldsymbol{v}, \delta_f^2 + \left(\frac{T\alpha}{1-\alpha} + \frac{(t_f - t_1)(t_f - t_2)}{(t_2 - t_1)(1-\alpha)^2}\right)\sigma_m^2\right).
\end{aligned}$$

Finally, we fill in this result and integrate with respect to $\boldsymbol{s}$ and simplify the resulting expression to obtain the displacement distribution over $[t_1, t_2]$. In the

following we use $\frac{\alpha}{1-\alpha} = \frac{t_1-t_s}{t_f-t_1}$. In the last step we use $\frac{t_2-t_1}{t_f-t_1} = \beta$ and $\frac{t_1-t_s}{T} = \alpha$.

$$\begin{aligned}
&\int_{\mathbb{R}^d} \phi(\boldsymbol{s}; \boldsymbol{\mu}_s, \delta_s^2)\phi\left(-\boldsymbol{s}; \frac{T}{t_2-t_1}\boldsymbol{v} - \boldsymbol{\mu}_f, \delta_f^2 + \left(\frac{T\alpha}{1-\alpha} + \frac{(t_f-t_1)(t_f-t_2)}{(t_2-t_1)(1-\alpha)^2}\right)\sigma_m^2\right) d\boldsymbol{s} \\
&= \left[\phi(\boldsymbol{\mu}_s, \delta_s^2) * \phi\left(\frac{T}{t_2-t_1}\boldsymbol{v} - \boldsymbol{\mu}_f, \delta_f^2 + \left(\frac{T\alpha}{1-\alpha} + \frac{(t_f-t_1)(t_f-t_2)}{(t_2-t_1)(1-\alpha)^2}\right)\sigma_m^2\right)\right](\boldsymbol{0}) \\
&= \phi\left(\boldsymbol{0}; \boldsymbol{\mu}_f - \boldsymbol{\mu}_s - \frac{T}{t_2-t_1}\boldsymbol{v}, \delta_s^2 + \delta_f^2 + \left(\frac{T(t_1-t_s)}{t_f-t_1} + \frac{(t_f-t_2)T^2}{(t_2-t_1)(t_f-t_1)}\right)\sigma_m^2\right) \\
&= \phi\left(\frac{T}{t_2-t_1}\boldsymbol{v}; \boldsymbol{\mu}_f - \boldsymbol{\mu}_s, \delta_s^2 + \delta_f^2 + \left(\frac{T(t_1-t_s)}{t_f-t_1} + \frac{(t_f-t_2)T^2}{(t_2-t_1)(t_f-t_1)}\right)\sigma_m^2\right) \\
&= \phi\Bigg(\boldsymbol{v}; \frac{t_2-t_1}{T}(\boldsymbol{\mu}_f - \boldsymbol{\mu}_s), \\
&\qquad \left(\frac{t_2-t_1}{T}\right)^2(\delta_s^2 + \delta_f^2) + \left(\frac{(t_2-t_1)^2(t_1-t_s)}{T(t_f-t_1)} + \frac{(t_f-t_2)(t_2-t_1)}{t_f-t_1}\right)\sigma_m^2\Bigg) \\
&= \phi\left(\boldsymbol{v}; \frac{t_2-t_1}{T}(\boldsymbol{\mu}_f - \boldsymbol{\mu}_s), \left(\frac{t_2-t_1}{T}\right)^2(\delta_s^2 + \delta_f^2) + \beta\left(\alpha(t_2-t_1) + (t_f-t_2)\right)\sigma_m^2\right).
\end{aligned}$$

From this distribution of the displacement, we obtain the velocity distribution by dividing by $t_2 - t_1$:

$$\begin{aligned}
\boldsymbol{V}(t_1, t_2) &= \frac{\boldsymbol{X}_2 - \boldsymbol{X}_1}{t_2 - t_1} \\
&\sim \mathcal{N}\left(\frac{\boldsymbol{\mu}_f - \boldsymbol{\mu}_s}{T}, \frac{\delta_s^2 + \delta_f^2}{T^2} + \beta\left(\frac{\alpha}{t_2-t_1} + \frac{t_f-t_2}{(t_2-t_1)^2}\right)\sigma_m^2\right).
\end{aligned} \tag{4}$$

We can simplify the last term of the variance using the definitions of α and β:

$$\begin{aligned}
&\beta\left(\frac{\alpha}{t_2-t_1} + \frac{t_f-t_2}{(t_2-t_1)^2}\right)\sigma_m^2 \\
&= \frac{t_2-t_1}{t_f-t_1}\left(\frac{t_1-t_s}{(t_f-t_s)(t_2-t_1)} + \frac{t_f-t_2}{(t_2-t_1)^2}\right)\sigma_m^2 \\
&= \frac{1}{t_f-t_1}\left(\frac{t_1-t_s}{t_f-t_s} + \frac{t_f-t_2}{(t_2-t_1)}\right)\sigma_m^2 \\
&= \frac{(t_1-t_s)(t_2-t_1) + (t_f-t_2)(t_f-t_s)}{(t_f-t_1)(t_f-t_s)(t_2-t_1)}\sigma_m^2 \\
&= \frac{(-t_1^2 + t_1(t_2+t_s) - t_2t_s) + (t_f^2 - t_f(t_2+t_s) + t_2t_s)}{(t_f-t_1)(t_f-t_s)(t_2-t_1)}\sigma_m^2 \\
&= \frac{(t_f-t_1)((t_f+t_1) - (t_2+t_s))}{(t_f-t_1)T(t_2-t_1)}\sigma_m^2 \\
&= \frac{(t_f-t_s) + (t_2-t_1)}{(t_f-t_s)(t_2-t_1)}\sigma_m^2 \\
&= \left(\frac{1}{t_f-t_s} + \frac{1}{t_2-t_1}\right)\sigma_m^2.
\end{aligned}$$

If we replace this into Equation 4, we obtain the following distribution for the velocity over $[t_2, t_1]$:

$$\boldsymbol{V}(t_1, t_2) \sim \mathcal{N}\left(\frac{\boldsymbol{\mu}_f - \boldsymbol{\mu}_s}{T}, \frac{\delta_s^2 + \delta_f^2}{T^2} + \left(\frac{1}{T} + \frac{1}{t_2 - t_1}\right)\sigma_m^2\right).$$

This result has intuitive appeal, since the mean velocity is exactly the velocity at which the mean location moves and identical to the velocity predicted by the linear movement model. One would expect the variance to grow as $t_2 - t_1$ decreases, and indeed the variance is inversely proportional to the length of the interval.

2.2 One measurement in the interval

Let $\boldsymbol{X}_s \sim \mathcal{N}\left(\boldsymbol{\mu}_s, \delta_s^2\right)$, $\boldsymbol{X}_i \sim \mathcal{N}\left(\boldsymbol{\mu}_i, \delta_i^2\right)$, and $\boldsymbol{X}_f \sim \mathcal{N}\left(\boldsymbol{\mu}_f, \delta_f^2\right)$ represent the positions at three consecutive times t_s, t_i and t_f where the location was sampled. If we want to determine the velocity distribution over a time interval $[t_1, t_2]$ such that $t_s \leq t_1 < t_i < t_2 \leq t_f$, the positions $\boldsymbol{X}_1, \boldsymbol{X}_2$ of an entity at these times cannot be regarded as independent, since they both depend on $\boldsymbol{X}_i$. Instead, we fix $\boldsymbol{X}_i$ at a position $\boldsymbol{x}$ and then use the Markov property of Brownian motion to derive a distribution for $\boldsymbol{X}_1$ and $\boldsymbol{X}_2$.

Define $\alpha := \frac{t_1 - t_s}{t_i - t_s}$, $\beta := \frac{t_2 - t_i}{t_e - t_i}$ and let $\sigma_{\boldsymbol{x}}^2(t)$ denote the variance at time t if the position at time t_i is fixed, i.e. the term that is proportional to δ_i^2 is removed.

$$\begin{aligned}
&f_{\boldsymbol{X}_2 - \boldsymbol{X}_1}(\boldsymbol{v}) \\
&= \int_{\mathbb{R}^d} f_{\boldsymbol{X}_i}(\boldsymbol{x})\, f_{\boldsymbol{X}_2 - \boldsymbol{X}_1}\left(\boldsymbol{v} \middle| \boldsymbol{X}_i = \boldsymbol{x}\right) d\boldsymbol{x} \\
&= \int_{\mathbb{R}^d} \phi\left(\boldsymbol{x}; \boldsymbol{\mu}_i, \delta_i^2\right) \cdot \phi\left(\boldsymbol{v}; \boldsymbol{\mu}_{\boldsymbol{x}}(t_2) - \boldsymbol{\mu}_{\boldsymbol{x}}(t_1), \sigma_{\boldsymbol{x}}^2(t_1) + \sigma_{\boldsymbol{x}}^2(t_2)\right) d\boldsymbol{x} \\
&= \int_{\mathbb{R}^d} \phi(\boldsymbol{x}; \boldsymbol{\mu}_i, \delta_i^2) \cdot \phi\left(\boldsymbol{v}; (1 - \beta - \alpha)\boldsymbol{x} + \beta\boldsymbol{\mu}_e - (1 - \alpha)\boldsymbol{\mu}_s, \sigma_{\boldsymbol{x}}^2(t_1) + \sigma_{\boldsymbol{x}}^2(t_2)\right) d\boldsymbol{x} \\
&= \int_{\mathbb{R}^d} \phi\left(\boldsymbol{x}; \boldsymbol{\mu}_i, \delta_i^2\right) \cdot \phi\left(\frac{\boldsymbol{v}}{1 - \beta - \alpha} - \boldsymbol{x}; \frac{\beta\boldsymbol{\mu}_e + (\alpha - 1)\boldsymbol{\mu}_s}{1 - \beta - \alpha}, \frac{\sigma_{\boldsymbol{x}}^2(t_1) + \sigma_{\boldsymbol{x}}^2(t_2)}{(1 - \beta - \alpha)^2}\right) d\boldsymbol{x} \\
&= \left[\phi\left(\boldsymbol{\mu}_i, \delta_i^2\right) * \phi\left(\frac{\beta\boldsymbol{\mu}_e + (\alpha - 1)\boldsymbol{\mu}_s}{1 - \beta - \alpha}, \frac{\sigma_{\boldsymbol{x}}^2(t_1) + \sigma_{\boldsymbol{x}}^2(t_2)}{(1 - \beta - \alpha)^2}\right)\right]\left(\frac{\boldsymbol{v}}{1 - \beta - \alpha}\right) \\
&= \phi\left(\frac{\boldsymbol{v}}{1 - \beta - \alpha}; \boldsymbol{\mu}_i + \frac{\beta\boldsymbol{\mu}_e + (\alpha - 1)\boldsymbol{\mu}_s}{1 - \beta - \alpha}, \delta_i^2 + \frac{\sigma_{\boldsymbol{x}}^2(t_1) + \sigma_{\boldsymbol{x}}^2(t_2)}{(1 - \beta - \alpha)^2}\right) \\
&= \phi\left(\boldsymbol{v}; (1 - \beta - \alpha)\boldsymbol{\mu}_i + \beta\boldsymbol{\mu}_e + (\alpha - 1)\boldsymbol{\mu}_s, (1 - \beta - \alpha)^2\delta_i^2 + \sigma_{\boldsymbol{x}}^2(t_1) + \sigma_{\boldsymbol{x}}^2(t_2)\right) \\
&= \phi\left(\boldsymbol{v}; \boldsymbol{\mu}(t_2) - \boldsymbol{\mu}(t_1), \sigma^2(t_1) + \sigma^2(t_2) - 2\alpha(1 - \beta)\delta_i^2\right).
\end{aligned}$$

We obtain the velocity distribution by dividing by $t_2 - t_1$:

$$\begin{aligned}
\boldsymbol{V}(t_1, t_2) &= \frac{\boldsymbol{X}_2 - \boldsymbol{X}_1}{t_2 - t_1} \\
&\sim \mathcal{N}\left(\frac{\boldsymbol{\mu}(t_2) - \boldsymbol{\mu}(t_1)}{t_2 - t_1}, \frac{\sigma^2(t_1) + \sigma^2(t_2) - 2\alpha(1 - \beta)\delta_i^2}{(t_2 - t_1)^2}\right).
\end{aligned}$$

Again, the mean velocity is the average rate at which the mean is displaced over $[t_1, t_2]$. The variance differs from the variance for independent positions by

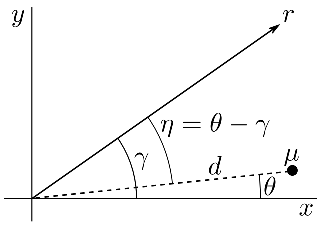

Figure 1: The variables involved in calculating the probability density of the direction of $\boldsymbol{X}$ at γ ($\boldsymbol{X}$ is on the ray r) when $\boldsymbol{X}$ has a circular normal distribution with mean $\boldsymbol{\mu}$ with polar coordinates (d, θ).

$-2\alpha(1-\beta)\delta_i^2$, which is largest when one of the endpoints is at t_i and goes to zero as an endpoint approaches t_s or t_f. This ensures that the distribution changes continuously as an interval of fixed length slides over a sampled trajectory.

2.3 Two or more measurements in the interval

Since we assume the positions at times of location measurements to be independent, $\boldsymbol{X}_1$ and $\boldsymbol{X}_2$ are independent if there are at least two measurements in $[t_1, t_2]$, so

$$\boldsymbol{X}_2 - \boldsymbol{X}_1 \sim \mathcal{N}\left(\boldsymbol{\mu}(t_2) - \boldsymbol{\mu}(t_1), \sigma^2(t_1) + \sigma^2(t_2)\right).$$

Thus we obtain the following expression for the velocity:

$$\begin{aligned}\boldsymbol{V}(t_1, t_2) &= \frac{\boldsymbol{X}_2 - \boldsymbol{X}_1}{t_2 - t_1} \\ &\sim \mathcal{N}\left(\frac{\boldsymbol{\mu}(t_2) - \boldsymbol{\mu}(t_1)}{t_2 - t_1}, \frac{\sigma^2(t_1) + \sigma^2(t_2)}{(t_2 - t_1)^2}\right).\end{aligned}$$

3 Speed and direction

Now that we have established that the velocity follows a circular normal distribution, we use this to derive expressions for the average speed and the average direction of movement over a time interval $[t_1, t_2]$.

Speed is the absolute value of velocity. It therefore has the distribution of the absolute value of a multivariate circular normal distribution. In the two-dimensional case this results in a Rice distribution [4]. Generally, in d-dimensional case the distribution is related to the noncentral chi distribution with d degrees of freedom. If $\boldsymbol{X} = (X_1, \ldots, X_d)$ has a multivariate circular normal distribution with mean $\boldsymbol{\mu} = (\mu_1, \ldots, \mu_d)$ and variance σ^2, then

$$Z = \frac{1}{\sigma}\sqrt{\sum_{i=1}^{d} X_i^2} = \frac{|\boldsymbol{X}|}{\sigma}$$

has a noncentral chi distribution with d degrees of freedom and noncentrality parameter

$$\lambda = \frac{1}{\sigma}\sqrt{\sum_{i=1}^{d} \mu_i^2} = \frac{|\boldsymbol{\mu}|}{\sigma}.$$

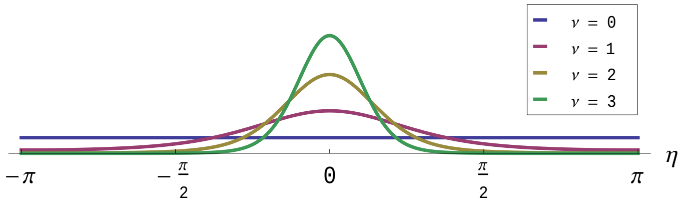

Figure 2: The distribution of the direction for various values of the noncentrality ν. The parameter η represents the angle between the direction of the mean velocity and the direction under consideration.

To derive the distribution of direction, it is convenient to work in polar coordinates. Let $d := |\boldsymbol{\mu}|$, $\theta := \text{atan2}(\boldsymbol{\mu})$ represent $\boldsymbol{\mu}$ in polar coordinates. See Figure 1 for an example of this situation. We consider direction only in two dimensions, the extension to higher dimensions is not directly obvious.

The probability density of a direction γ may be considered to be the integral of the distribution of $\boldsymbol{V}$ over an infinitesimally narrow cone with its apex at the origin. Let $\boldsymbol{u}_\gamma$ be the unit vector at an angle γ from the positive x-axis. This gives the following expression for the probability density function of the direction Γ:

$$\begin{aligned} f_\Gamma(\gamma) &= \int_0^\infty \phi\left(r\boldsymbol{u}_\gamma; \boldsymbol{\mu}, \sigma^2\right) r dr \\ &= \int_0^\infty \frac{1}{2\pi\sigma^2} e^{\frac{-|r\boldsymbol{u}_\gamma - \boldsymbol{\mu}|^2}{2\sigma^2}} r dr. \end{aligned}$$

Now, rotate everything by an angle $-\gamma$ around the origin, such that $r\boldsymbol{u}_\gamma = r\boldsymbol{u}_0 = (r, 0)$ runs over the positive x-axis. The new polar coordinates for $\boldsymbol{\mu}$ are now $(d, \eta) := (d, \theta - \gamma)$, so we get the following expression for $f(\gamma)$.

$$\begin{aligned} f_\Gamma(\gamma) &= \int_0^\infty \frac{1}{2\pi\sigma^2} e^{\frac{-|r\boldsymbol{u}_\gamma - \boldsymbol{\mu}|^2}{2\sigma^2}} r dr \\ &= \frac{1}{2\pi\sigma^2} \int_0^\infty e^{\frac{-(r - d\cos\eta)^2 - (0 - d\sin\eta)^2}{2\sigma^2}} r dr \\ &= \frac{1}{2\pi\sigma^2} \int_0^\infty e^{\frac{-(r^2 - 2rd\cos\eta + d^2\cos^2\eta + d^2\sin^2\eta)}{2\sigma^2}} r dr \\ &= \frac{1}{2\pi\sigma^2} \int_0^\infty e^{\frac{-(r^2 - 2rd\cos\eta + d^2)}{2\sigma^2}} r dr. \end{aligned}$$

Evaluating this integral using Mathematica 8 [5] gives

$$f_\Gamma(\gamma) = \frac{e^{-\frac{\nu^2}{2}}}{2\pi} + \frac{\nu\cos\eta}{2\sqrt{2\pi}} e^{\frac{\nu^2(\cos^2\eta - 1)}{2}} \left(1 + \text{erf}\left(\frac{\nu\cos\eta}{\sqrt{2}}\right)\right), \tag{5}$$

where $\nu := \frac{d}{\sigma}$ is the *noncentrality* of the velocity distribution and $\text{erf}(z) = 2/\sqrt{\pi}\int_0^z e^{-t^2} dt$ is the Gauss error function. See Figure 2 for some examples of this distribution. For $\nu = 0$, the direction is uniformly distributed as expected. For positive ν, there is a preference for $\eta = 0$, i.e. the velocity is in the direction of the mean. This preference gets stronger as ν increases.

4 Spatial distributions of speed

To obtain a spatial distribution of speed, we fix the position at a time t and determine the mean speed over a time interval $[t + \Delta t_s, t + \Delta t_f]$. The mean speed at position $\boldsymbol{x}$ is obtained by averaging this value over t, weighted by the probability that $\boldsymbol{X}_t = \boldsymbol{x}$. Since $\boldsymbol{X}_t$ is fixed, the position at a time before t and a time after t are independent by the Markov property of Brownian motion. Thus, if $\Delta t_s \leq 0$ and $\Delta t_f \geq 0$ we can determine the distribution of $\boldsymbol{X}_{t+\Delta t_s}$ and $\boldsymbol{X}_{t+\Delta t_f}$ independently and derive the velocity distribution from those.

4.1 Distribution of position with fixed $\boldsymbol{X}_t$

We will now derive the distribution of $\boldsymbol{X}_{t+\Delta t}$ for the case $\Delta t > 0$. The case $\Delta t < 0$ is symmetric and will not be discussed here. If $\Delta t = 0$, then $\boldsymbol{X}_{t+\Delta t} = \boldsymbol{X}_t \sim \mathcal{N}(\boldsymbol{x}, 0)$ has a degenerate distribution and is thus known exactly.

Assume that the measurements directly before and after t were obtained at the times t_{i-1} and t_i with locations $\boldsymbol{X}_{(i-1)} \sim \mathcal{N}\left(\boldsymbol{\mu}_{i-1}, \delta_{i-1}^2\right)$ and $\boldsymbol{X}_{(i)} \sim \mathcal{N}\left(\boldsymbol{\mu}_i, \delta_i^2\right)$, respectively. We will use α here as parameter for the Brownian bridge between t_{i-1} and t_i (as in Equation 1). The distribution of $\boldsymbol{X}_{(i)}$ is influenced by the location of $\boldsymbol{x}$. We use Bayes' theorem to obtain the distribution of $\left(\boldsymbol{X}_{(i)} \middle| \boldsymbol{X}_t = \boldsymbol{x}\right)$:

$$
\begin{aligned}
&f_{\boldsymbol{X}_{(i)}}\left(\boldsymbol{q} \middle| \boldsymbol{X}_t = \boldsymbol{x}\right) \\
&= \frac{f_{\boldsymbol{X}_t}\left(\boldsymbol{x} \middle| \boldsymbol{X}_{(i)} = \boldsymbol{q}\right) f_{\boldsymbol{X}_{(i)}}(\boldsymbol{q})}{f_{\boldsymbol{X}_t}(\boldsymbol{x})} \\
&= \frac{\phi\left(\boldsymbol{x}; (1-\alpha)\boldsymbol{\mu}_{i-1} + \alpha\boldsymbol{q}, (1-\alpha)^2\delta_{i-1}^2 + (t_i - t_{i-1})\alpha(1-\alpha)\sigma_m^2\right)\right) \phi\left(\boldsymbol{q}; \boldsymbol{\mu}_i, \delta_i^2\right)}{f_{\boldsymbol{X}_t}(\boldsymbol{x})} \\
&= \frac{\phi\left(\boldsymbol{q}; \frac{\alpha-1}{\alpha}\boldsymbol{\mu}_{i-1} + \frac{\boldsymbol{x}}{\alpha}, \left(\frac{1-\alpha}{\alpha}\right)^2 \delta_{i-1}^2 + \frac{(t_i - t_{i-1})(1-\alpha)}{\alpha}\sigma_m^2\right)\right) \phi\left(\boldsymbol{q}; \boldsymbol{\mu}_i, \delta_i^2\right)}{f_{\boldsymbol{X}_t}(\boldsymbol{x})}.
\end{aligned} \tag{6}
$$

Now let

$$
\begin{aligned}
\boldsymbol{\mu}_q &:= \frac{\alpha - 1}{\alpha}\boldsymbol{\mu}_{i-1} + \frac{\boldsymbol{x}}{\alpha}, \\
\sigma_q^2 &:= \left(\frac{1-\alpha}{\alpha}\right)^2 \delta_{i-1}^2 + \frac{(t_i - t_{i-1})(1-\alpha)}{\alpha}\sigma_m^2
\end{aligned} \tag{7}
$$

be the parameters of the first normal distribution obtained in the equation above. The product of two normal densities is again a Gaussian function, although it will have to be scaled to be a density function. In particular,

$$
\phi\left(\boldsymbol{q}; \boldsymbol{\mu}_q, \sigma_q^2\right) \phi\left(\boldsymbol{q}; \boldsymbol{\mu}_i, \delta_i^2\right) = \phi\left(\boldsymbol{q}; \frac{\boldsymbol{\mu}_q \delta_i^2 + \boldsymbol{\mu}_i \sigma_q^2}{\sigma_q^2 + \delta_i^2}, \frac{\sigma_q^2 \delta_i^2}{\sigma_q^2 + \delta_i^2}\right) \phi\left(\boldsymbol{0}; \boldsymbol{\mu}_q - \boldsymbol{\mu}_i, \sigma_q^2 + \delta_i^2\right).
$$

We can rewrite the second density on the right hand side, that is independent of $\boldsymbol{q}$ and acts as a scaling factor, to obtain:

$$\begin{aligned}
&\phi\left(\mathbf{0};\boldsymbol{\mu}_q-\boldsymbol{\mu}_i,\sigma_q^2+\delta_i^2\right)\\
&=\phi\left(\mathbf{0};\frac{\alpha-1}{\alpha}\boldsymbol{\mu}_{i-1}+\frac{\boldsymbol{x}}{\alpha}-\boldsymbol{\mu}_i,\left(\frac{1-\alpha}{\alpha}\right)^2\delta_{i-1}^2+\frac{(t_i-t_{i-1})(1-\alpha)}{\alpha}\sigma_m^2+\delta_i^2\right)\\
&=\phi\left(\boldsymbol{x};(1-\alpha)\boldsymbol{\mu}_{i-1}+\alpha\boldsymbol{\mu}_i,(1-\alpha)^2\delta_{i-1}^2+\alpha^2\delta_i^2+(t_i-t_{i-1})\alpha(1-\alpha)\sigma_m^2\right)\\
&=f_{\boldsymbol{X}_t}\left(\boldsymbol{x}\right).
\end{aligned}$$

Inserting this in Equation 6 yields

$$\begin{aligned}
&f_{\boldsymbol{X}_{(i)}}\left(\boldsymbol{q}\middle|\boldsymbol{X}_t=\boldsymbol{x}\right)\\
&=\frac{\phi\left(\boldsymbol{q};\frac{\alpha-1}{\alpha}\boldsymbol{\mu}_{i-1}+\frac{\boldsymbol{x}}{\alpha},\left(\frac{1-\alpha}{\alpha}\right)^2\delta_{i-1}^2+\frac{(t_i-t_{i-1})(1-\alpha)}{\alpha}\sigma_m^2\right)\phi\left(\boldsymbol{q};\boldsymbol{\mu}_i,\delta_i^2\right)}{f_{\boldsymbol{X}_t}\left(\boldsymbol{x}\right)}\\
&=\frac{\phi\left(\boldsymbol{q};\frac{\boldsymbol{\mu}_q\delta_i^2+\boldsymbol{\mu}_i\sigma_q^2}{\sigma_q^2+\delta_i^2},\frac{\sigma_q^2\delta_i^2}{\sigma_q^2+\delta_i^2}\right)f_{\boldsymbol{X}_t}\left(\boldsymbol{x}\right)}{f_{\boldsymbol{X}_t}\left(\boldsymbol{x}\right)}\\
&=\phi\left(\boldsymbol{q};\frac{\boldsymbol{\mu}_q\delta_i^2+\boldsymbol{\mu}_i\sigma_q^2}{\sigma_q^2+\delta_i^2},\frac{\sigma_q^2\delta_i^2}{\sigma_q^2+\delta_i^2}\right),
\end{aligned}\tag{8}$$

with $\boldsymbol{\mu}_q$ and σ_q^2 as defined in Equation 7. So the location at the time of measurement is still normally distributed, although the parameters are influenced by the location and time of $\boldsymbol{X}_t$. Let $\boldsymbol{\mu}_{i^*}$ and $\sigma_{i^*}^2$ be the parameters of this conditional distribution.

4.2 Distribution of $\boldsymbol{X}_{t+\Delta t}$

Now that we have the distribution at the time of the measurement that comes after $\boldsymbol{X}_t$, we can derive the distribution of $\boldsymbol{X}_{t+\Delta t}$. How this is derived depends on the number of measurements that were obtained between t and $t+\Delta t$.

4.2.1 No measurements between t and $t+\Delta t$.

We can define a smoothed Brownian bridge starting at $\boldsymbol{X}_t=\boldsymbol{x}$ and ending at $\boldsymbol{X}_{(i)}$, conditioned on $\boldsymbol{X}_t=\boldsymbol{x}$. Let $\beta:=\frac{\Delta t}{t_i-t}$. Then,

$$\boldsymbol{X}_{t+\Delta t}\sim\mathcal{N}\left((1-\beta)\boldsymbol{x}+\beta\boldsymbol{\mu}_{i^*},\beta^2\sigma_{i^*}^2+(t_i-t)\beta(1-\beta)\sigma_m^2\right).$$

4.2.2 One measurement between t and $t+\Delta t$.

We can derive the distribution of $\boldsymbol{X}_{t+\Delta t}$ using a Brownian bridge running from this conditioned value of $\boldsymbol{X}_{(i)}$ to $\boldsymbol{X}_{(i+1)}$, where $\boldsymbol{X}_{(i+1)}$ is the location at the time of the measurement that follows $\boldsymbol{X}_{(i)}$. Let $\beta:=\frac{t+\Delta t-t_i}{t_{i+1}-t_i}$. Then,

$$\boldsymbol{X}_{t+\Delta t}\sim\mathcal{N}\left((1-\beta)\boldsymbol{\mu}_{i'}+\beta\boldsymbol{\mu}_{i+1},(1-\beta)^2\sigma_{i'}^2+\beta^2\delta_{i+1}^2+(t_i-t)\beta(1-\beta)\sigma_m^2\right).$$

4.2.3 Multiple measurements between t and $t+\Delta t$.

In this case, $\boldsymbol{X}_{t+\Delta t}$ is independent from $\boldsymbol{X}_t$ since we assume independence of the measurements. Thus, $\boldsymbol{X}_{t+\Delta t}$ is distributed as described by the position distribution for the BBMM in Equation 2.

4.3 Conditioned speed distribution

Now that we know the distribution at any time conditioned on $\boldsymbol{X}_t$, we can determine this position distribution for two separate times and derive the distribution of the velocity from that. As discussed at the beginning of Section 4, we can straightforwardly derive the velocity distribution over $[t + \Delta t_s, t + \Delta t_f]$ from the position distributions at the endpoints of the interval, since they can be treated as independent if $\Delta t_s \leq 0$ and $\Delta t_f \geq 0$. Let $\boldsymbol{X}_{t+\Delta t_s} \sim \mathcal{N}\left(\boldsymbol{\mu}_s, \sigma_s^2\right)$ and $\boldsymbol{X}_{t+\Delta t_f} \sim \mathcal{N}\left(\boldsymbol{\mu}_f, \sigma_f^2\right)$. The velocity over the interval $[t + \Delta t_s, t + \Delta t_f]$ conditioned on $\boldsymbol{X}_t = \boldsymbol{x}$ is given by

$$\boldsymbol{V}_{\boldsymbol{x};t}\left(t + \Delta t_s, t + \Delta t_f\right) = \frac{\boldsymbol{X}_{t+\Delta t_f} - \boldsymbol{X}_{t+\Delta t_s}}{\Delta t_f - \Delta t_s} \sim \mathcal{N}\left(\frac{\boldsymbol{\mu}_f - \boldsymbol{\mu}_s}{\Delta t_f - \Delta t_s}, \frac{\sigma_s^2 + \sigma_f^2}{\left(\Delta t_f - \Delta t_s\right)^2}\right).$$

As discussed in Section 3, when the velocity is normally distributed, speed (its absolute value) is Rice distributed (in two dimensions). In order to determine a spatial distribution of speeds we compute a weighted average of the mean speed over time, keeping the interval over which the speed is computed fixed relative to the time at which the position is fixed. The weight is given by $f_{\boldsymbol{X}_t}(\boldsymbol{x})$. That is,

$$S(\boldsymbol{x}) := \frac{1}{\int f_{\boldsymbol{X}_t}(\boldsymbol{x})\, dt} \int f_{\boldsymbol{X}_t}(\boldsymbol{x})\, \mathbb{E}\left[\left|V_{\boldsymbol{x},t}(t + \Delta t_s, t + \Delta t_f)\right|\right] dt. \tag{9}$$

The interval of integration is chosen such that the integrand can be evaluated for every point in the interval. That is, location data must be available at times t, $t + \Delta t_s$ and $t + \Delta t_f$.

Author details

[1]Department of Mathematics and Computer Science, Technical University Eindhoven, Eindhoven, The Netherlands. [2]Faculty of Mathematics, Ruhr-Universität Bochum, Bochum, Germany. [3]Computational Geo-Ecology, Institute for Biodiversity and Ecosystem Dynamics, University of Amsterdam, Amsterdam, The Netherlands. [4]Department of Evolutionary and Environmental Biology, The University of Haifa, Haifa, Israel. [5]Institut de Biologie, Université de Neuchâtel, Neuchâtel, Switzerland. [6]Anthropological Institute & Museum, University of Zurich, Zurich, Switzerland.

References

1. Bullard, F.: Estimating the home range of an animal: A Brownian bridge approach. Master's thesis, The University of North Carolina (1999)
2. Pozdnyakov, V., Meyer, T., Wang, Y.-B., Yan, J.: On modeling animal movements using Brownian motion with measurement error. Ecology **95**, 247–253 (2014). doi:10.1890/13-0532.1
3. Buchin, K., Sijben, S., Arseneau, T.J.M., Willems, E.P.: Detecting movement patterns using Brownian bridges. In: Proceedings of the 20th International Conference on Advances in Geographic Information Systems, pp. 119–128. ACM, New York, NY, USA (2012). doi:10.1145/2424321.2424338. http://doi.acm.org/10.1145/2424321.2424338
4. Kobayashi, H., Mark, B.L., Turin, W.: Probability, Random Processes, and Statistical Analysis: Applications to Communications, Signal Processing, Queueing Theory and Mathematical Finance. Cambridge University Press, Cambridge (2011)
5. Wolfram Research, Inc.: Mathematica Edition: Version 8.0. Wolfram Research, Inc., Champaign, Illinois (2010)
