# Supplementary material for: Deriving movement properties and the effect of the environment from the Brownian bridge movement model in monkeys and birds
Source: Mov Ecol. 2015 Jun 15;3(1):18. doi: 10.1186/s40462-015-0043-8 (PMC4466871; doi:10.1186/s40462-015-0043-8)
Supplement: Additional file 5 — R scripts (first study). R scripts used in the first case study. [file 40462_2015_43_MOESM5_ESM.zip › movementAnalysis_0.7.7.zip!/movementAnalysis/html/00Index.html]

R: Analysis of trajectory data using linear or Brownian motion
model

# Analysis of trajectory data using linear or Brownian motion model

---

## Documentation for package ‘movementAnalysis’ version 0.7.7

- DESCRIPTION file.

## Help Pages

|  |  |
| --- | --- |
| barplot.encounterDuration | Draw a barplot representing the duration of encounters. |
| contour.utilizationDistribution | Plot or draw contours for utilization distributions. |
| contourPolygons | Compute home range polygons from utilization distribution |
| ddirection | Compute the (distribution of) movement direction. |
| ddistance | Compute distance statistics |
| diffusionCoefficient-method | Class '"MoveBB"' |
| diffusionCoefficient-method | Class '"MoveBBStack"' |
| direction | Compute the (distribution of) movement direction. |
| displacement.direction | Collect various statistics about individual bursts |
| displacement.distance | Collect various statistics about individual bursts |
| distance | Compute distance statistics |
| djl | Collect various statistics about individual bursts |
| dspeed | Get statistics about speed |
| encounter | Compute distance statistics |
| encounterDistribution | Spatial distribution of encounters |
| encounterDuration | Compute duration of encounters between groups |
| encounterDurationById | Compute duration of encounters between groups |
| encounterIntervals | Compute distance statistics |
| monkey.data | Trajectory data of two monkeys over two days |
| monkey.tr | Trajectory data of two monkeys over two days |
| MoveBB-class | Class '"MoveBB"' |
| moveBB-method | Create a 'MoveBB' object |
| moveBB-methods | Create a 'MoveBB' object |
| moveBBStack | Create a 'MoveBBStack' object |
| MoveBBStack-class | Class '"MoveBBStack"' |
| mu.speed | Get statistics about speed |
| pdirection | Compute the (distribution of) movement direction. |
| pdistance | Compute distance statistics |
| plot.utilizationDistribution | Plot or draw contours for utilization distributions. |
| position | Evaluate position parameters at the given times |
| position-method | Class '"MoveBB"' |
| position-method | Class '"MoveBBStack"' |
| pspeed | Get statistics about speed |
| qdistance | Compute distance statistics |
| qspeed | Get statistics about speed |
| speed | Get statistics about speed |
| speedDistribution | Compute average speed at each location |
| speedDistribution-method | Class '"MoveBB"' |
| speedDistribution-method | Class '"MoveBBStack"' |
| split-method | Class '"MoveBBStack"' |
| utilizationDistribution | Compute utilization distribution for a trajectory |
| utilizationDistribution-method | Class '"MoveBB"' |
| utilizationDistribution-method | Class '"MoveBBStack"' |
| utilizationDistribution.byID | Compute utilization distribution for a trajectory |
| velocity | Compute distribution parameters for velocity |
| velocity-method | Class '"MoveBB"' |
| velocity-method | Class '"MoveBBStack"' |
| [-method | Class '"MoveBB"' |
| [-method | Class '"MoveBBStack"' |
| [[.utilizationDistribution | Compute utilization distribution for a trajectory |
